# Lymphocytic Esophagitis Presenting With Food Impaction

**DOI:** 10.7759/cureus.42873

**Published:** 2023-08-02

**Authors:** Majd B Aboona, Kelli Kosako Yost, Paul Muńa Aguon, Brian M Fung, Rakesh Nanda

**Affiliations:** 1 Department of Internal Medicine, University of Arizona College of Medicine, Phoenix, USA; 2 Division of Gastroenterology, Department of Internal Medicine, University of Arizona College of Medicine, Phoenix, USA; 3 Department of Gastroenterology and Hepatology, Carl T. Hayden Phoenix Veterans Administration (VA) Medical Center, Phoenix, USA

**Keywords:** esophagus, food impaction, esophagitis, dysphagia, lymphocytic esophagitis

## Abstract

Lymphocytic esophagitis (LyE) is a rare diagnosis made on esophageal biopsy whose pathogenesis is poorly understood. Since its appearance in the literature 15 years ago, it still remains an enigma due to its low prevalence. In this case report, a 71-year-old male presented with an episode of acute dysphagia due to food impaction. Urgent endoscopy was performed to fragment the food bolus. Repeat endoscopy showed a stricture, and lymphocytic esophagitis was found on esophageal biopsy. A proton pump inhibitor (PPI) was initiated with symptomatic improvement. With its increasing prevalence, lymphocytic esophagitis should be on the differential for causes of dysphagia.

## Introduction

Esophagitis is a common disorder with many different etiologies, including reflux; infectious; eosinophilic; and, most recently, lymphocytic types. Lymphocytic esophagitis (LyE) is an emerging, yet not well understood, condition that shares certain features with gastroesophageal reflux disease (GERD) and eosinophilic esophagitis (EoE) [1]. All three types of esophagitis present with similar symptoms, most commonly dysphagia. Esophageal biopsy is the gold standard for diagnosis, but because the pathogenesis is not well understood, treatment options are limited and mostly reflect current treatment recommendations for GERD [2]. The clinical manifestations of this condition include esophageal dysphagia and typical symptoms of GERD. Additional symptoms include abdominal/chest pain, nausea/vomiting, and odynophagia [2]. Esophageal rings, webs, erythema, nodularity, and mucosal fragility can be found in LyE, but the most common finding is an endoscopy with normal findings [3,4]. This condition is currently defined solely on histological criteria, specifically the presence of >20 intraepithelial lymphocytes per high-power field [5].

## Case presentation

**Figure 1:**
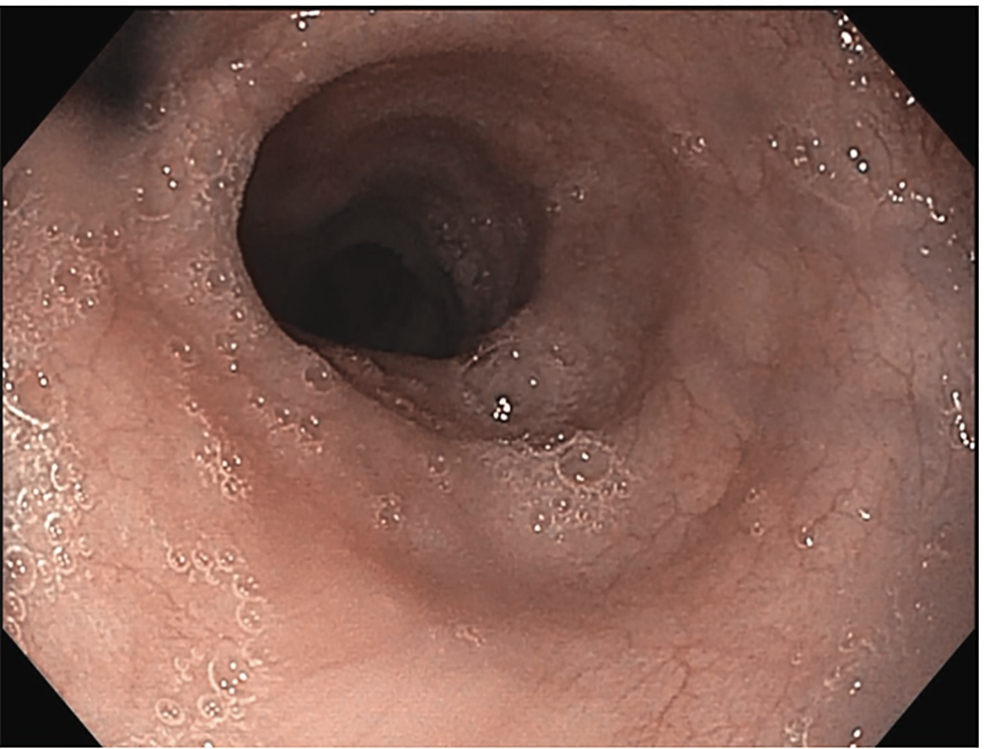
EGD image showing esophageal mucosa EGD: endogastroduodenoscopy

**Figure 2:**
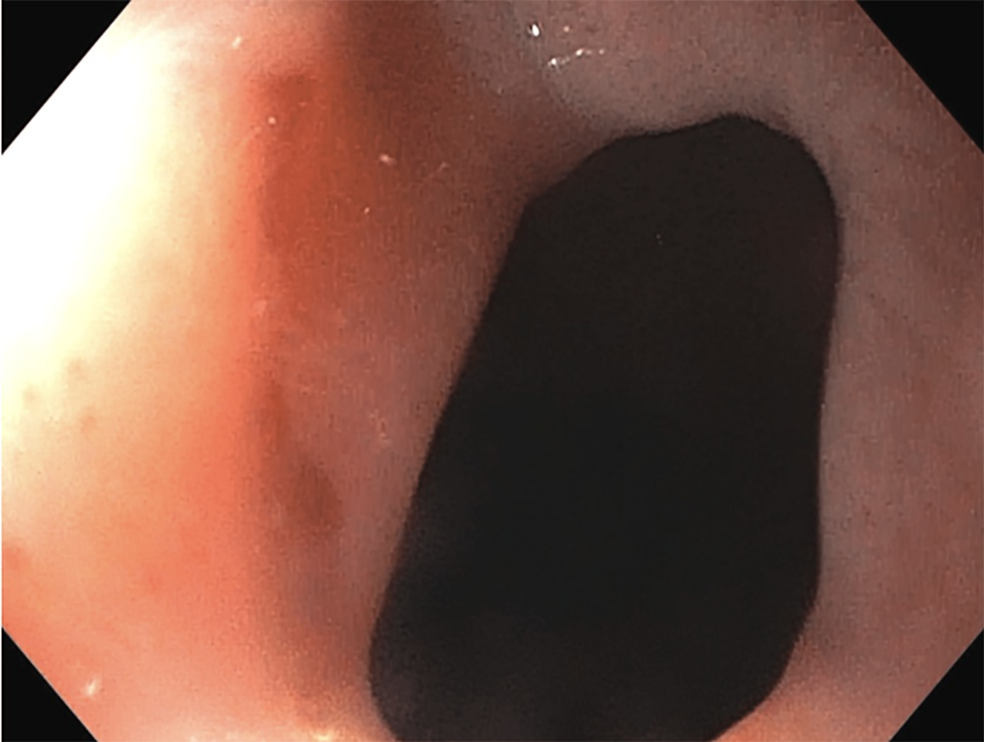
EGD image showing esophageal stricture present at the distal esophagus EGD: endogastroduodenoscopy

The patient is a 71-year-old male with a past medical history of hypertension, tobacco use disorder, and alcohol use disorder who presented after experiencing an episode of acute dysphagia to solids and liquids with concern for food impaction. Prior to this episode, the patient had no history of dysphagia or reflux symptoms. Urgent endogastroduodenoscopy (EGD) was performed, which showed a large food bolus at the distal esophagus/gastroesophageal (GE) junction. This was fragmented and pushed distally into the stomach by Roth Net (Steris, Mentor, OH). Several ulcers were present in the lower esophagus, and biopsies taken from the gastroesophageal junction and stomach showed mild erosive gastropathy and intestinal metaplasia without dysplasia. *Helicobacter pylori* testing was negative. No stricture was noted in the esophagus. Repeat EGD was performed eight weeks later, and a distal esophageal stricture was noted and was dilated with a balloon dilator to 15 mm. Biopsies taken of the proximal and distal esophagus showed squamous mucosa with features of lymphocytic esophagitis. The patient was placed on omeprazole twice daily and reported the improvement of his symptoms and the resolution of dysphagia at one-week and six-month follow-ups. The patient reported compliance with omeprazole. One month later, a barium swallow was completed that showed no recurrence of esophageal stricture (Figures 1, 2).

## Discussion

Lymphocytic esophagitis was first described in the literature by Rubio et al. in 2006 [6]. The prevalence of this pathology in the literature has increased in recent years, possibly due to the volume of esophageal biopsies taken during EGD or due to increasing recognition of the condition by pathologists [2,4]. In one large study of over 129,000 patients, lymphocytic esophagitis was discovered in approximately 0.1% of esophageal biopsies [3].

There have been several retrospective studies on lymphocytic esophagitis, although these were of low power due to the rarity of the disorder. Of these studies, 52%-63% of those affected were females with the median age of diagnosis being 51-56 years [2,3]. This distinguishes lymphocytic esophagitis from eosinophilic esophagitis, as EoE is more prevalent in younger males with a median age of 43, with 66% male predominance [3].

Lymphocytic esophagitis is distinguished by the presence of a lymphocytic infiltrate on biopsy, usually with greater than or equal to 20-40 intraepithelial lymphocytes per high-power field with only rare neutrophils or eosinophils [2,3]. Other characteristics may be present and include patchy, peripapillary lymphocytic infiltrates with associated spongiotic edema [3]. The most common presenting symptoms include dysphagia, chest pain, abdominal pain, and odynophagia [2,3]. Dysphagia was by far the most common presenting symptom and is present in 66%-71% of patients [2,3]. These symptoms are very similar to the presentation of EoE and GERD.

Appearance on the endoscopy of lymphocytic esophagitis is highly variable and ranges from normal-appearing mucosa in 21%-30% of the time to anomalies such as esophagitis in 16%-33% of patients and esophageal rings in 19%-30% of patients [2,3]. This patient specifically presented with a food impaction in the setting of lymphocytic esophagitis. Food impaction and its association with LyE have also been studied and were present in up to 9% of patients in one study [7].

The pathogenesis of LyE is not well known. Some infer that the presence of lymphocytes is due to an inflammatory process [4]. One study on ablation therapy in Barrett's esophagus proposed that LyE is an inflammatory response that occurs due to esophageal injury, as demonstrated by the increase in lymphocytes post-ablation found 280 days later [8]. Our patient had LyE diagnosed post-endoscopic therapy for a food impaction, resulting in stricture. This could support the theory that esophageal trauma/injury could be associated with the development of LyE. Others have inferred that LyE may exist on a spectrum with GERD, as there have been studies showing an increased number of lymphocytes in GERD with one study showing that 5% of esophageal biopsies in patients with GERD also met the diagnosis for LyE [1]. Yet, another postulation is that LyE may have an autoimmune component as it has been associated with other conditions, including hypothyroidism, inflammatory bowel disease, asthma, and seasonal allergies [2]. There has also been a proposed association with smoking, which is also a risk factor in our patient [3,8]. This is a common theme with certain autoimmune conditions, such as rheumatoid arthritis, in that genetically predisposed individuals encounter an environmental trigger, such as tobacco, and develop the condition.

In regard to treatment, many of the modalities used in GERD have been utilized, especially proton pump inhibitors (PPIs). The treatment of lymphocytic esophagitis includes symptomatic management such as proton pump inhibitors, swallowed topical steroids, and endoscopic dilation [9,10]. In one prospective study with a follow-up of patients over three years, most had resolution of their symptoms with the use of a PPI [2]. This may be due to the anti-inflammatory effects of PPIs or the possible concomitant association of LyE with GERD [4]. In this patient specifically, PPI initiation resulted in symptom improvement.

## Conclusions

This case illustrates a case of dysphagia and food impaction with underlying lymphocytic-predominant esophageal pathology. Due to the rarity of this condition, the pathogenesis and evidence-based treatment have yet to be discovered. Increasing prevalence brings increasing recognition on esophageal biopsy, which could illicit a unique pathophysiology of lymphocytic esophagitis and therefore an optimal treatment strategy.
